# *Brucella abortus* Traverses Brain Microvascular Endothelial Cells Using Infected Monocytes as a Trojan Horse

**DOI:** 10.3389/fcimb.2018.00200

**Published:** 2018-06-14

**Authors:** María C. Miraglia, Ana M. Rodriguez, Paula Barrionuevo, Julia Rodriguez, Kwang S. Kim, Vida A. Dennis, M. Victoria Delpino, Guillermo H. Giambartolomei

**Affiliations:** ^1^Instituto de Inmunología, Genética y Metabolismo, Consejo Nacional de Investigaciones Científicas y Técnicas, Universidad de Buenos Aires, Buenos Aires, Argentina; ^2^Instituto de Medicina Experimental, Consejo Nacional de Investigaciones Científicas y Técnicas-Academia Nacional de Medicina, Buenos Aires, Argentina; ^3^Division of Pediatric Infectious Diseases, Department of Pediatrics, Johns Hopkins University School of Medicine, Baltimore, MD, United States; ^4^Center for NanoBiotechnology Research and Department of Biological Sciences, Alabama State University, Montgomery, AL, United States

**Keywords:** blood-brain barrier, endothelial cells, monocytes, *Brucella abortus*, neurobrucellosis

## Abstract

Neurobrucellosis is an inflammatory disease caused by the invasion of *Brucella* spp. to the central nervous system (CNS). The pathogenesis of the disease is not well characterized; however, for *Brucella* to gain access to the brain parenchyma, traversing of the blood-brain barrier (BBB) must take place. To understand the CNS determinants of the pathogenesis of *B. abortus*, we have used the *in vitro* BBB model of human brain microvascular endothelial cells (HBMEC) to study the interactions between *B. abortus* and brain endothelial cells. In this study, we showed that *B. abortus* is able to adhere and invade HBMEC which was dependent on microtubules, microfilaments, endosome acidification and de novo protein synthesis. After infection, *B. abortus* rapidly escapes the endosomal compartment of HBMEC and forms a replicative *Brucella*-containing vacuole that involves interactions with the endoplasmic reticulum. Despite the ability of *B. abortus* to invade and replicate in HBMEC, the bacterium was unable by itself to traverse HBMEC, but could traverse polarized HBMEC monolayers within infected monocytes. Importantly, infected monocytes that traversed the HBMEC monolayer were a bacterial source for *de novo* infection of glial cells. This is the first demonstration of the mechanism whereby *B. abortus* is able to traverse the BBB and infect cells of the CNS. These results may have important implications in our understanding of the pathogenesis of neurobrucellosis.

## Introduction

Invasion of the central nervous system (CNS) is a severe event during the course of many infectious diseases which can lead to severe neurological sequelae (Kim, 2003; Saez-Llorens and McCracken, 2003; van de Beek et al., 2006). The way in which microbes interact with and cross the blood-brain barrier (BBB) to gain access to the brain parenchyma is a key event in the pathogenesis of CNS infections. Considerable efforts have been made in understanding the mechanisms whereby bacterial pathogens with a predominantly extracellular life cycle invade the CNS from the bloodstream (Kim, 2002, 2008; Nassif et al., 2002). However, the mechanisms used by intracellular bacteria to enter the CNS are less well-established.

Brucellosis is primarily a disease of domestic and wild animals that can be transmitted to humans, in whom it affects several organs and tissues, given rise to various clinical manifestations (Young, 1995; Pappas et al., 2005). Invasion of the nervous system by *Brucella* results in an inflammatory disorder called neurobrucellosis. Mostly, it affects the CNS, and has a fateful prognosis (McLean et al., 1992; Giambartolomei et al., 2008). Neurobrucellosis may manifest as meningoencephalitis, brain abscesses, meningovascular disease, demyelinating syndromes, and myelitis (Bouza et al., 1987; McLean et al., 1992; Giambartolomei et al., 2008).

It is generally believed that CNS involvement in neurobrucellosis occurs by hematogenous dissemination. Yet, for *Brucella* spp. the exact mechanism by which the bacterium leaves the bloodstream and enters the CNS remains unclear. Since *Brucella* smooth species have developed diverse mechanisms to survive intracellularly, particularly within macrophages (Celli, 2006), BBB translocation within infected phagocytes (the so-called Trojan horse mechanism) has been postulated as a possible mechanism of CNS invasion by *Brucella* spp. (Drevets et al., 2004). However, this possibility has not been confirmed experimentally. Besides, *Brucella* spp. can invade and replicate in diverse non-phagocytic cells (Pizarro-Cerdá et al., 1998; García Samartino et al., 2010; Ferrero et al., 2011; Scian et al., 2011, 2012; Starr et al., 2012; Arriola Benitez et al., 2013), and therefore transcellular invasion of brain microvascular endothelial cells might be another possible route of CNS invasion by *Brucella*. Alternatively, but less probable (Drevets et al., 2004), *Brucella* organisms could use paracellular migration between barrier cells. Irrespective of the mechanism used by the bacterium, it is evident that once it reaches the CNS it triggers a pathological pro-inflammatory response (Giambartolomei et al., 2008; García Samartino et al., 2010; Miraglia et al., 2013, 2016; Rodriguez et al., 2017).

The intracellular life and immune responses of *B. abortus* have been extensively studied *in vitro* and *in vivo* (Baldwin and Goenka, 2006; Celli, 2006; Pappas, 2010; von Bargen et al., 2012); yet many aspects of the pathophysiology of brucellosis, and particularly that of neurobrucellosis remain elusive (Baldi and Giambartolomei, 2013a,b; de Figueiredo et al., 2015), in part due to the absence of an appropriate and easy-to-handle animal model that mimics all the hallmarks of the human disease. Despite these limitations, our group has recently unraveled some aspects of the immunopathology of neurobrucellosis (García Samartino et al., 2010; Miraglia et al., 2013, 2016; Rodriguez et al., 2017), but the mechanism by which *B. abortus* enters the CNS still remains unknown.

In this paper we demonstrate the capacity of *B. abortus* to adhere, invade and replicate in human brain microvascular endothelial cells (HBMEC), revealing the eukaryotic mechanisms for the invasion process. Using a culture transwell model of the BBB, we then examined the migratory capacity of *B. abortus* to traverse HBMEC and the mechanism involved in this process. The results of our study are presented herein.

## Materials and methods

### Ethics statement

Human monocytes were isolated from blood of healthy adult donors in agreement with the guidelines of the the Ethical Committee on Clinical Investigation of the School of Pharmacy and Biochemistry of the University of Buenos Aires (Protocol N° 0048885/2016). All adult blood donors provided their informed consent prior to the study in accordance with the Declaration of Helsinki (2013) of the World Medical Association. Animal experiments were approved by the Committee of Care and Use of laboratory animals of the School of Medicine, University of Buenos Aires (Permit Number: 358/2015).

### Bacteria

*Brucella abortus* S2308, DsRed-expressing *B. abortus* 2308 (kindly provided by Diego Comerci, UNSAM University, Argentina), *Escherichia coli* HB101 and *Citrobacter freundii* were grown overnight in 10 ml tryptic soy agar supplemented with yeast extract (Merck) with constant agitation (150 rpm) at 37°C. Bacteria were collected by centrifugation at 6,000 × g for 15 min at 4°C and washed twice in 10 ml of phosphate-buffered saline (PBS). Bacterial numbers were assessed by comparing the optical densities at 600 nm with a standard curve obtained in our laboratory, and the inocula were prepared as described previously (Miraglia et al., 2013). Manipulations of live *Brucella* were performed in biosafety level 3 facilities located at the Instituto de Investigaciones Biomédicas en retrovirus y SIDA (Buenos Aires, Argentina).

### HBMEC culture

Immortalized HBMEC were obtained as previously described (Stins et al., 2001). HBMEC were cultured in 75-ml tissue cultures flasks in RPMI 1640 medium supplemented with 10% NuSerum IV (Becton Dickinson, Bedford, MA), heat-inactivated 10% FBS (Life Technologies, Grand Island, NY), 1% modified Eagle's medium nonessential amino acids (Life Technologies), L-glutamine (2 mM), sodium pyruvate (1 mM), MEM vitamin solution (Life Technologies), and penicillin-streptomycin (complete medium).

### Glial cell culture

Astrocytes and microglia (~95% of purity) were established from primary mixed glial cultures obtained from the forebrain of 1- to 3-d-old C57BL/6 mice according to previously published procedures (García Samartino et al., 2010).

### Monocyte purification

Peripheral blood mononuclear cells were isolated from blood of healthy donors by density gradient centrifugation using Ficoll-Hypaque (GE Healthcare). Isolation of monocytes (CD14^+^ cells) was accomplished using CD14 microbeads (Miltenyi Biotec). Purified monocytes (>98% purity as determined by flow cytometry) were resuspended in RPMI 1,640 supplemented with 10% heat-inactivated FBS for subsequent studies. Viability of cells, as measured by trypan blue exclusion test, was more than 95% in all the experiments.

### Infection

Infection of cells was performed for 2 h in medium containing no antibiotics followed by extensive washing of cells to remove non-internalized bacteria. Cells were then maintained or not at different times in the presence of 50 μg/ml streptomycin and 100 μg/ml gentamicin to kill all remaining extracellular bacteria. HBMEC were infected at different multiplicities of infection (MOI), while monocytes and glial cells were infected at a MOI of 100. Infected cells were lysed with 0.1% (v/v) Triton X-100 in H_2_O after PBS washing, and serial dilutions of lysates were plated onto tryptone soya broth agar plates to enumerate colony forming units (CFU).

### Internalization inhibition assay

Infection assays were carried out as described above except that HBMEC were pretreated with complete medium containing the inhibitors cytochalasin D, colchicine, cycloheximide or monensin (Sigma-Aldrich) at the indicated concentrations. HBMEC were incubated with each compound for 1 h at 37°C prior to infection and the inhibitor was maintained in the culture throughout the experiment. After culture, cells were lysed with 0.1% (v/v) Triton X-100 to evaluate intracellular invasion by quantification of CFU. Control cells were treated with an equivalent amount of vehicle lacking the active compound. The vehicles used to dissolve colchicine, cycloheximide, cytochalasin D, and monensin were, respectively, complete medium, dimethyl sulfoxide (DMSO), and ethanol. The concentrations of DMSO and ethanol were always below 0.05% in the culture medium.

### Transcytosis experiments

Monolayers of polarized HBMEC were established by culturing 2 × 10^4^ cells per insert on Transwell plates [6.5-mm diameter and a 3 μm pore size membrane insert previously treated with rat tail collagen (50 mg/ml with 1% acetic acid; BD Biosciences)], which were further neutralized in a closed container enriched in vapors of ammonium hydroxide (Transwell Clear Polyester Membrane insert; Corning-Costar, Acton, MA). After 5 days, when cellular confluence was reached, *B. abortus* (1 × 10^8^ bacteria) were added to the apical side of the insert. Samples were collected from the basolateral chambers at the indicated incubation times and were plated to quantify CFU. An equivalent volume of medium removed was replaced with fresh medium in each culture. Simultaneously passive diffusion of horseradish peroxidase was measured as an indication of monolayer integrity. Noninvasive *E. coli HB101* was used as a negative control and *C. freundii* was used as a control of transcellular migration (each at 1 × 10^8^ bacteria). Alternatively, monocytes previously infected with *B. abortus* at a MOI of 100 (1 × 10^5^) were added to the upper chamber of the monolayer of HBMEC. All plates were incubated at 37°C in 5% CO_2_ for 4 h, after which transmigrated cells in the lower chamber were counted on a hemocytometer and CFU were quantified after cell lysis.

### Confocal microscopy

For subcellular localization experiments, HBMEC (5 × 10^4^) that were plated onto glass coverslips of 12 mm in diameter were infected with DSRed-*B. abortus* (MOI of 100) for different time-points. After infection, cells were fixed with 4% paraformaldehyde (PFA), permeabilized with 0.1% saponin and blocked with 10% FBS for 30 min. Co-localization markers were detected using mAbs specific for LAMP-2 (late endosomes/lysosomes) and calnexin (ER) (both from BD Biosciences); and EEA-1 (early endosomes) (Synaptic Systems), followed by Alexa 488-labeled secondary Ab (Invitrogen). To evaluate monocytes as a bacterial source for de novo infection of glial cells, primary microglia or astrocytes (3 × 10^4^ cells) were cultured on glass coverslips for 24 h. Afterwards, astrocytes were co-cultured for 24 or 48 h with DS Red *Brucella*-infected monocytes that transmigrated (for 4 h) or not through a monolayer of HBMEC growing in the transwell system described above. Co-cultures were fixed with 4% PFA, permeabilized with 0.125% Triton X-100 and blocked with 5% FBS. Microglia were stained with Isolectin-B4-Biotin (Vector Laboratories) followed by CyTM2 Streptavidin (Jackson ImmunoResearch). Astrocytes were stained with anti-GFAP (Biogenex) followed by Alexa 488-labeled secondary Ab (Invitrogen). In all cases TO-PRO®-3 (Invitrogen) was used for nuclear staining. Slides were mounted with PolyMount (Polysciences) and analyzed using FV-1000 confocal microscope with an oil immersion Plan Apochromatic 60¥ NA1.42 objective (Olympus).

### Statistical analysis

Statistical analysis was performed with one-way ANOVA, followed by Bonferroni Post Test using GraphPad Prism 4.0 software. Data is represented as the mean ± SEM.

## Results

### *B. abortus* adheres, invades, and replicates in brain microvascular endothelial cells

**Figure 1:**
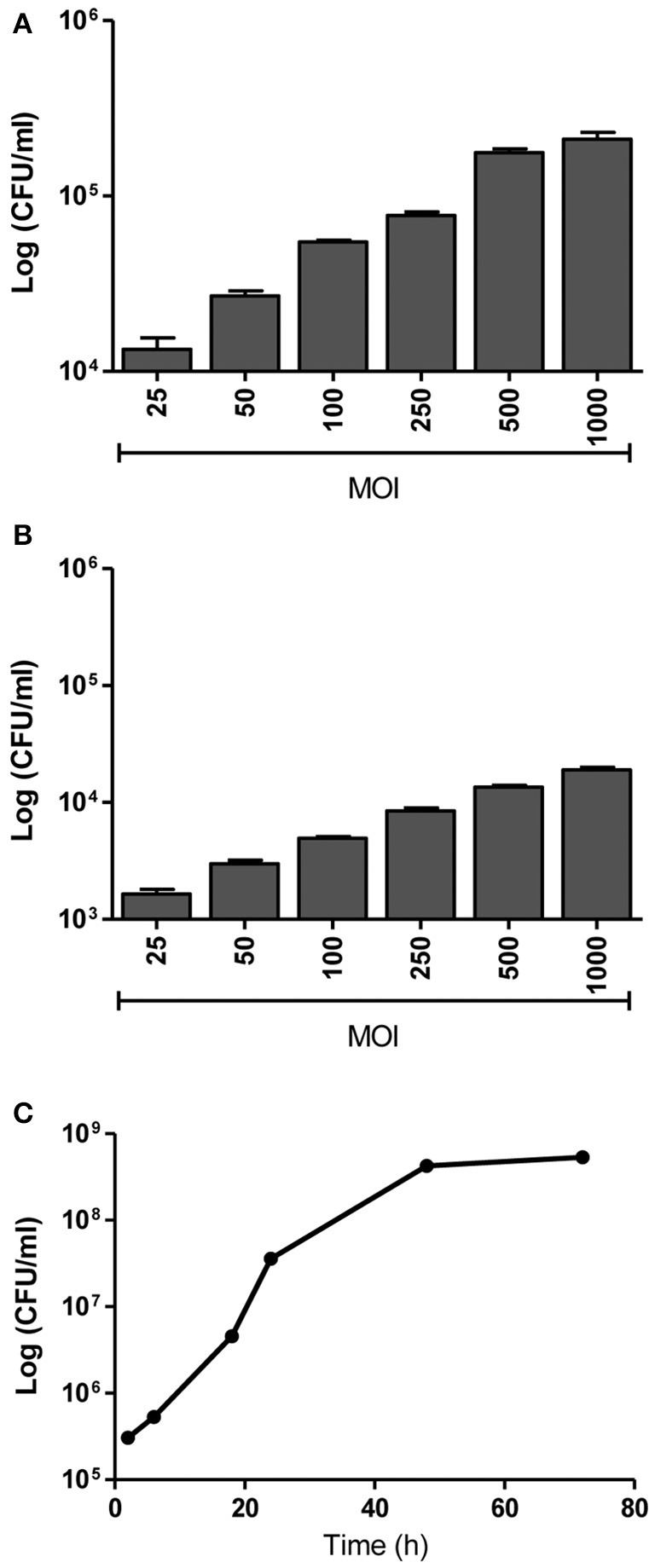
*B. abortus* adheres, invades and replicates in HBMEC. HBMEC were incubated for 2 h with *B. abortus* 2308 at the indicated MOI. Adhered bacteria were calculated by the difference of CFU/ml between HBMEC treated or not with antibiotics **(A)**. Internalized bacteria were determined by enumerating the CFU within cells at 24 h post-infection **(B)**. Bacterial replication was determined by counting CFU at different times after infecting HBMEC at a MOI of 100 **(C)**. Bars represent the mean ± SEM of duplicates.

**Figure 2:**
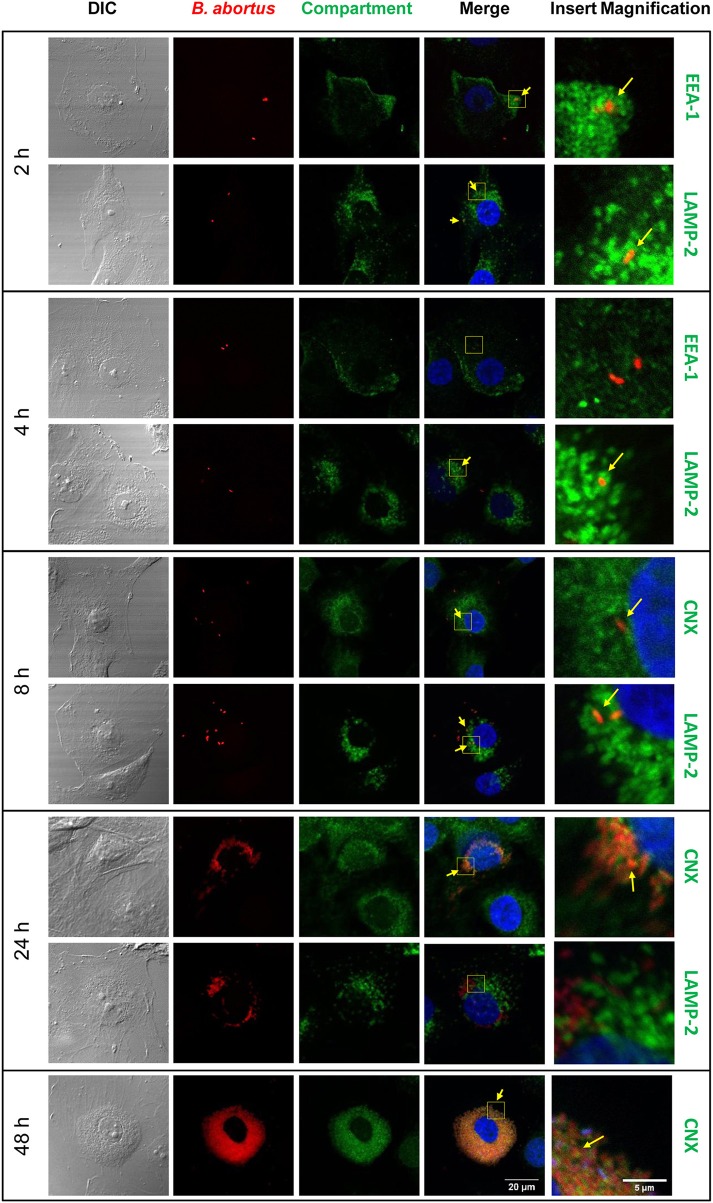
Subcellular localization of *B. abortus* during HBMEC infection. *B. abortus* subcellular localization was evaluated in HBMEC infected with DSRed *B. abortus* at MOI 100 at 2, 4, 8, 24, and 48 h post-infection. Subcellular compartments were determined with mAbs specific for EEA1 (early endosomes), LAMP-2 (late endosomes/lysosomes), and calnexin (ER) followed by secondary Ab Alexa 488-labeled (green staining). TO-PRO-3® (blue staining) was used to counter-stain nuclei. Yellow arrows show co-localization. EEA-1: early endosome antigen 1, LAMP-2: lysosome-associated membrane protein 2; CNX: calnexin. Data shown are from a representative experiment of three performed.

We have previously demonstrated the capacity of *B. abortus* to invade and replicate in human umbilical vein endothelial cells (HUVEC) (Ferrero et al., 2011) as a model of endothelial cells from peripheral vasculature. Thus, we decided to evaluate the ability of *B.abortus* to interact with HBMEC as a model of brain microvasculature (Stins et al., 2001). We first determined the capacity of *B. abortus* to adhere to HBMEC by incubating them for 2 h with *B. abortus*. Then, cells were washed to eliminate unbound bacteria after which wells were treated with or without antibiotics to kill extracellular bacteria. Cells lysates were obtained and plated in each case, and adherence was calculated as the difference in CFU between wells not treated and those treated with antibiotics. *B. abortus* adhered to HBMEC in a MOI-dependent fashion (Figure 1A). CFU quantification in wells treated with antibiotics revealed that *B. abortus* invades and replicates in HBMEC (Figures 1B,C); thereby corroborating and extending our previous findings (Miraglia et al., 2016). To study the subcellular localization of *B. abortus* in infected HMBEC we designed experiments in which cells were infected with DSRed-*B. abortus* and sub-cellular compartments were labeled with specific primary mAbs followed by Alexa 488-labeled (green) secondary Ab and then analyzed by confocal microscopy at different time-points post-infection. As early as 2 h post-infection most bacteria were excluded from the endosomal compartment and only ~34% of bacteria were found in compartments labeled for the early endosomal antigen 1 (EEA-1) (Figure 2). At 4 h post-infection, most of the *Brucella*-containing vacuoles (BCVs) were LAMP-2+. Later on, LAMP-2+ BCVs progressively decreased and at 24 h after infection the majority of bacteria were enclosed within calnexin+ (endoplasmic reticulum marker) vacuoles and they remained positive for calnexin up to 48 h post-infection (Figure 2). These results indicate that in HBMEC *B. abortus* invades and rapidly escapes the endocytic compartment and replicates within calnexin positive vacuoles.

### *B. abortus* is actively internalized by HBMEC

**Figure 3:**
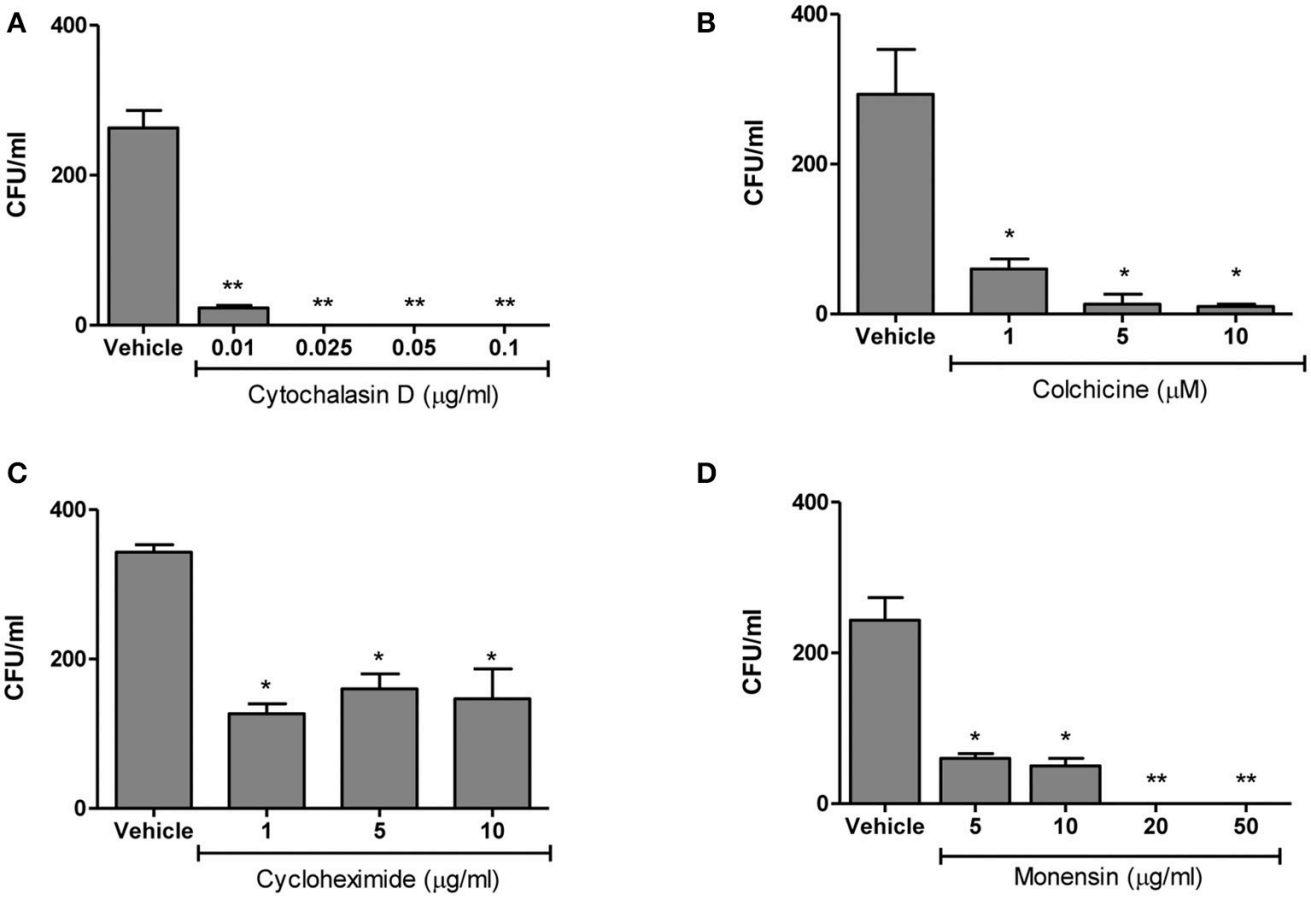
*B. abortus* is actively internalized by HBMEC. HBMEC were pre-incubated with different inhibitors for 1 h at 37°C. Inhibitors were kept throughout the infection. After 24 h cells were lysed and CFU enumerated **(A–D)**. Bars represent the mean ± SEM of duplicates. *p < 0.1, **p < 0.01 vs. vehicle-treated HBMEC. Data shown are from a representative experiment of three performed.

We next evaluated the eukaryotic cellular components necessary for *B. abortus* invasion by investigating the effects of various eukaryotic inhibitors on *B. abortus* invasion of HBMEC. The function of the actin-based cytoskeleton in *B. abortus* invasion was investigated by employing cytochalasin D, an agent that causes microfilament depolymerization in eukaryotic cells. To examine the participation of microtubules, colchicine—a microtubule-destabilizing agent—was employed. The role of *de novo* eukaryotic protein synthesis in *B. abortus* invasion was determined in assays performed with cycloheximide-treated HBMEC. Last, to determine the role of endosome acidification in *B. abortus* invasion, the inhibitor monensin (a cationic ionophore that increases the pH of intracellular vacuoles was used in invasion assays (Badger et al., 1999). HBMEC were pre-treated with different concentrations of the inhibitors and then infected with *B. abortus* and intracellular invasion was determined by intracellular CFU quantification. All inhibitors significantly (*p* < 0.01) inhibited *B. abortus* invasion of HBMEC (Figure 3). Taken together, these results suggest that *B. abortus* invasion of HBMEC depends on microtubules, de novo protein synthesis, microfilaments and endosome acidification.

### *B. abortus* is unable to traverse polarized HBMEC monolayers transcellularly or paracellularly

**Figure 4:**
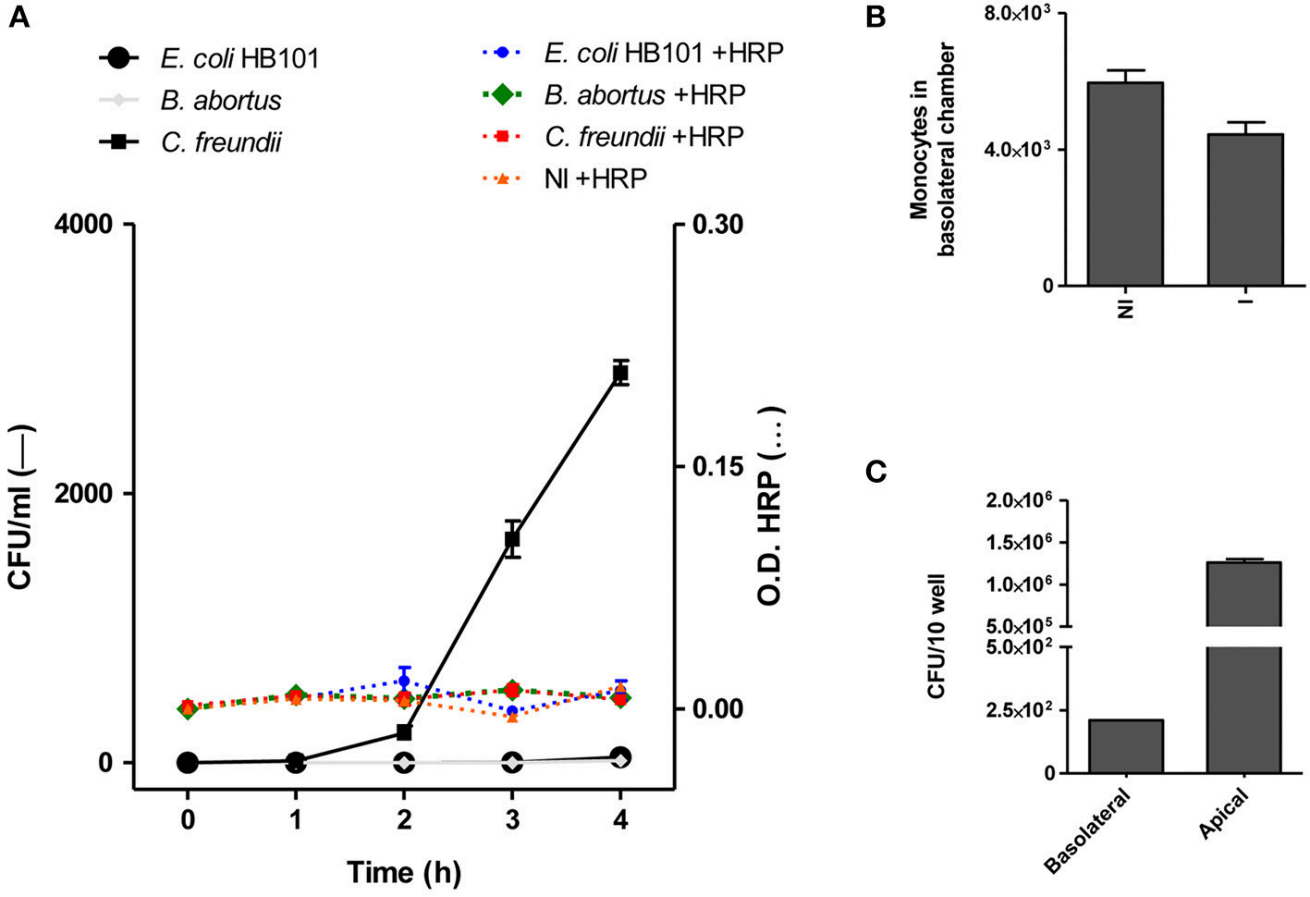
*B. abortus* transcytoses polarized HBMEC monolayers within infected monocytes. HBMEC were grown to confluence on Transwell filters as described in materials and methods. *B. abortus* 2308, *E. coli* HB101, and *C. freundii* (bacteria) were added to the apical side. Samples were collected from the basolateral chambers at indicated incubation times and were plated for CFU quantification. HRP (1/500) was added together with bacteria and its activity was evaluated in basolateral chamber as a control of monolayer integrity **(A)**. *B. abortus*-infected monocytes (1 × 10^5^) were added to the upper chamber of the monolayer of HBMEC to evaluate bacterial transmigration. After 4 h transmigrated cells were counted in the bottom well using a hemocytometer **(B)** and the bacterial quantification (CFU) was calculated in cell lysates of 10 wells in the lower chamber **(C)**. Bars shown represent the mean ± SEM of duplicates. Data shown are from a representative experiment of three performed. OD, Optical density.

Bacteria of the genus *Brucella* have developed several mechanisms to invade and persist within cells, not only macrophages but also non-phagocytic cells (Pizarro-Cerdá et al., 1998; Celli, 2006). Thus, taking into account their ability to invade HBMEC, we investigated whether *B. abortus* was able to traverse the brain microvascular endothelium. Diverse researchers have previously performed experiments using transwells as a model system to study bacterial and fungal transcytosis through intact polarized HBMEC monolayer constituting the BBB (Nizet et al., 1997; Ring et al., 1998; Badger et al., 1999; Jong et al., 2001; Chang et al., 2004). Based on this, in the present study, polarized HBMEC monolayers were established on transwell inserts followed by addition of *B. abortus* to the upper chamber of the transwell (apical side of HBMEC). At different times, the presence of the bacterium in the bottom chamber (basolateral side of HBMEC) was determined by CFU counting to ascertain the bacterial crossing through the HBMEC monolayer. *E. coli* HB101 was used as a noninvasive bacterium control, while *C. freundii* was employed as a positive control of a bacterium that is able to traverse HBMEC monolayer transcellularly (Badger et al., 1999). Horseradish peroxidase (HRP) was simultaneously added to the apical chamber, and its activity was evaluated together with the bacterial presence in the basolateral chamber as an indicator of monolayer integrity and passive diffusion. Under these experimental conditions *C. freundii* was able to traverse the polarized monolayer in a time-dependent manner, whereas noninvasive HB101 showed no HBMEC traversal. On the other hand, *B. abortus* was unable to traverse the HBMEC monolayers (Figure 4A). Levels of HRP diffusion were negligible and similar to all conditions tested indicating that the integrity of the monolayers was not altered and that *C. freundii* migration took place by a transcellular mechanism, as reported (Badger et al., 1999; Figure 4A). In the absence of HBMEC, both HRP and *B. abortus* easily crossed the transwell filter, indicating that the pore size of the transwell filters (3-μm) does not form a barrier for either HRP or *B. abortus* (data not shown). These results indicate that *B. abortus* is unable to traverse HBMEC monolayers by a transcellular or paracellular mechanism.

### *B. abortus* traverses polarized HBMEC monolayers within infected monocytes

We next investigated whether *B. abortus* could traverse the brain endothelium within infected monocytes. For this, *B. abortus*-infected or uninfected monocytes were added to the apical chamber of polarized HBMEC in the transwell plate and after 4 h, the presence of monocytes in the bottom chamber (basolateral side of HBMEC) was evaluated. At the same time, by intracellular CFU counting, the presence of *B. abortus* was determined within monocytes. This experiment was conducted in the presence of antibiotics, thus CFU correspond exclusively to monocyte-dwelling intracellular bacteria. Although there was no difference in the number of infected and uninfected monocytes that migrated through the HBMEC monolayer (Figure 4B), we recovered *B. abortus* in the basolateral side of the monolayer as a direct consequence of monocyte-carrying *Brucella* that migrated through HBMEC (Figure 4C). Taken together, these results suggest a Trojan horse mechanism of *B. abortus* traversal across the BBB.

### Infected monocytes are bacterial source for *de novo* infection of glial cells

**Figure 5:**
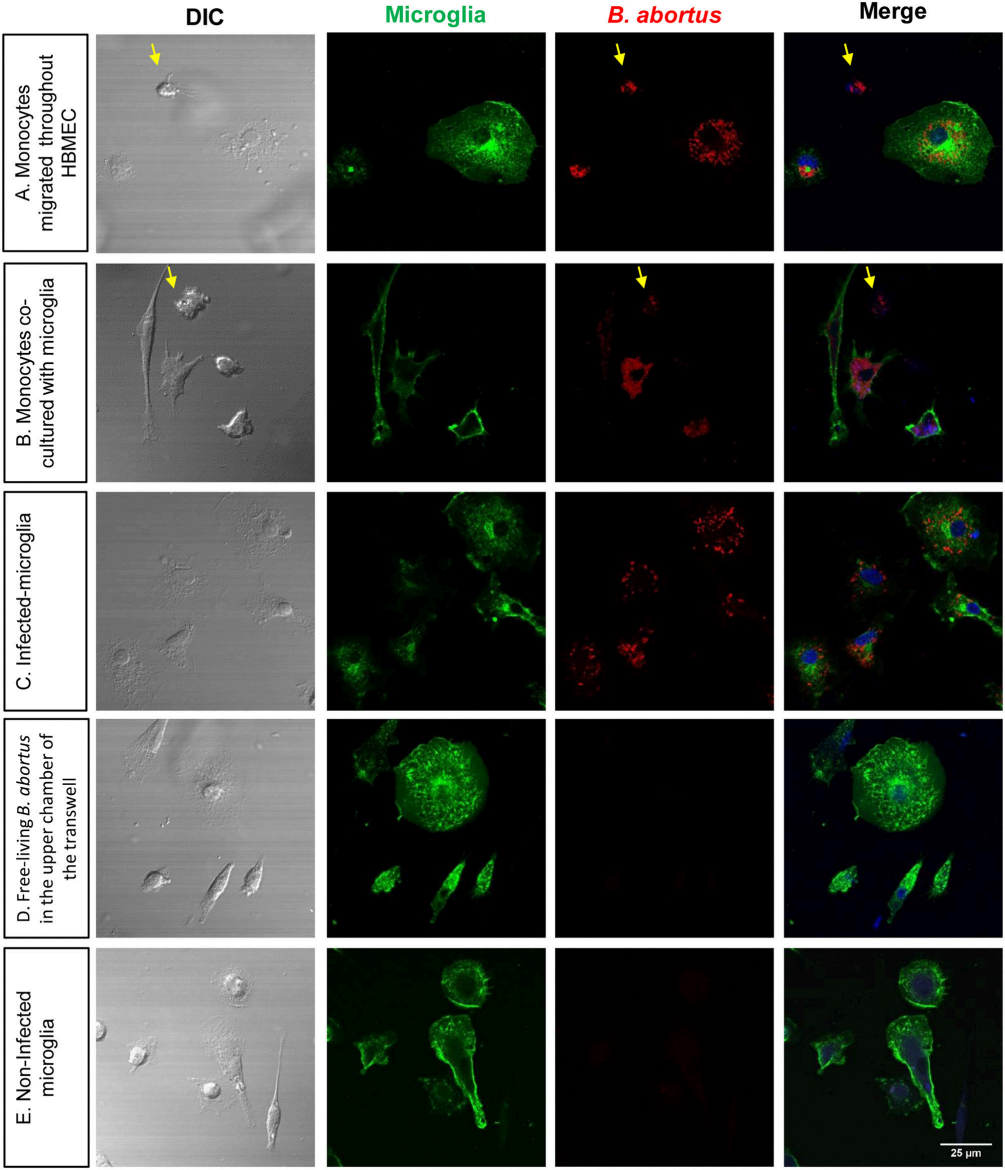
Infected monocytes are bacterial source for de novo infection of astrocytes. Microglia were co-cultured for 24 h with DS-red *Brucella*-infected monocytes that transmigrate the monolayer of HBMEC **(A)**. Infected monocytes were also directly co- cultured with uninfected microglia **(B)**. As a positive control of infection, microglia were directly infected with DS-red *Brucella* **(C)**. As negative control, free-living *B. abortus* was incubated in the upper chamber of a transwell, and microglia and astrocytes in the bottom chamber **(D)**. Non-infected microglia were also cultured as a negative control **(E)**. Microglia were labeled with isolectin-B4 mAb followed by CyTM2 Streptavidin (green staining). TO-PRO-3® (blue staining) was used to counter-stain nuclei. Arrows show monocytes in the culture.

We have contended that once *B. abortus* enters the CNS it infects glial cells triggering a pathological pro-inflammatory response (García Samartino et al., 2010; Miraglia et al., 2016; Rodriguez et al., 2017). Since we demonstrated that *B. abortus* traverses HBMEC monolayers inside infected monocytes, we examined how it would infect glial cells if surviving inside monocytes. Thus, we investigated if monocyte-dwelling *Brucella* could be a source of *de novo* infection for glial cells. For this, 24 h DSRed-*B. abortus*-infected monocytes that had traversed the HBMEC monolayer were added to primary cultures of microglia or astrocytes and incubated for 24 or 48 h. After culture, DSRed-*B. abortus* presence inside glial cells was determined by microscopy. At the same time, microglia and astrocytes were co-cultured with infected monocytes or directly infected with DSRed-*B. abortus*, to serve as positive controls. As negative control, free-living *B. abortus* was incubated in the upper chamber of a transwell, and microglia and astrocytes in the bottom chamber. Bacterial egress and subsequent glial cell infection was corroborated by the presence of DS Red-*B. abortus* inside microglia and astrocytes. Corroborating our premise, infected monocytes that traverse the HBMEC monolayer were able to infect microglia and astrocytes. The same was true if infected monocytes were co-cultured with glial cells. On the contrary, free-living *B. abortus* was unable to traverse HBMEC monolayers and infect glial cells (Figure 5 and data not shown). These results suggest that *B. abortus* invasion of the CNS within infected monocytes could generate infection in glial cells.

## Discussion

The CNS is protected from the environment by the skull, the spine, the meninges, the cerebrospinal fluid and the BBB. BBB ensures the well-being of the neural milieu by restricting the flow of blood-borne ions, molecules and cells into the neural tissue, protecting it from any microorganisms or toxins within the blood circulation (Rubin and Staddon, 1999). However, many pathogens have evolved complex mechanisms to target this line of defense, resulting in either the microbial invasion of cells constitutive of the BBB, or the disruption of barrier integrity leading to invasion of the brain parenchyma.

Although *Brucella* organisms preferentially live in professional phagocytes, these bacteria are also able to infect and replicate in other cell types such as osteocytes, osteoblasts, synoviocytes, hepatic stellate cells, hepatocytes, and glial cells (Delpino et al., 2010; García Samartino et al., 2010; Scian et al., 2011, 2012; Arriola Benitez et al., 2013; Pesce Viglietti et al., 2016). *B. abortus* is also able to survive within epithelial cells (Comerci et al., 2001; Ferrero et al., 2009); particularly in colonic epithelial cells (Ferrero et al., 2012; Czibener et al., 2016) that also form another specialized barrier that restricts the access of potential hazards to the organism: the gut immune barrier (Daneman and Rescigno, 2009). Moreover, the ability of *Brucella* organisms to survive in endothelial cells of the peripheral vasculature has also been described (Ferrero et al., 2011). Our present results show the capacity of *B. abortus* to adhere, infect and replicate in HBMEC adding new evidence on the capacity of *B. abortus* to survive within non-phagocytic cells such as HBMEC.

We have focused on interactions of *B. abortus* and the BBB and try to shed light on the infectious process of endothelial cells of brain microvasculature. With the use of specific inhibitors we elucidated that *B. abortus* internalization to HBMEC involves microtubules, actin microfilaments, *de novo* protein synthesis and endosome acidification. Actin restructuration has been recently implicated as a key factor during *B. abortus* cellular invasion, internalization and intracellular trafficking in epithelial cells. In these cells actin microfilaments were demonstrated to be critical in the formation of the replicative vacuole (Czibener et al., 2016). In line with these, *B. abortus* internalization in intestinal and alveolar epithelial cells also depends on actin microfilaments and microtubules, and partially on *de novo* protein synthesis (Ferrero et al., 2009, 2010). *B. abortus* internalization also involves microtubules and the activation of GTPases of the Rho subfamily in Hela cells (Guzmán-Verri et al., 2001). Thus, our results add more evidence indicating that in non-phagocytic cells *Brucella* exploits an active mechanism that involves different eukaryotic components.

Our results provided evidence that *B. abortus* is unable to traverse HBMEC transcellularly despite its capacity to infect these cells. Probably, the reasons for such contradiction could reside at the root of the biological cycle of this particular bacterium. Most microorganisms that are able to cross brain endothelial cells by the transcellular mechanism are observed intracellularly within membrane-bound endocytic vacuoles (Nizet et al., 1997; Ring et al., 1998; Badger et al., 1999; Jong et al., 2001; Chang et al., 2004). Conversely, when infecting a cell *B. abortus* escapes the endocytic vacuolar pathway to establish a replicative BCV by co-opting elements of the endoplasmic reticulum (Celli et al., 2003). In fact, as early as 2 h after HBMEC infection, *B. abortus*-containing vacuoles lose markers of early endosomes and rapidly gain markers of late endosomes and endoplasmic reticulum. After 24 h of infection, the majority of bacteria were enclosed within calnexin positive vacuoles (ER marker) and remained there even after 48 h of infection.

It is important to consider that, for most extracellular bacteria, neuroinvasion takes place in the context of systemic disease and often is linked to high bacteremia (Kim, 2003). Interestingly, it has been demonstrated that upon infection of mice with *B. melitensis*, the bacteria can either remain free-living or associated with red cells in the blood (Vitry et al., 2014). These same authors pointed out that as infection progresses the majority of bacteria are associated with leukocytes, agreeing with the brucellosis research field which vindicate the intracellular nature of *Brucella* organisms during blood dissemination. Thus, and as demonstrated here, if infection of brain endothelial cells could occur *in vivo* this phenomenon would not be relevant in CNS infection since free living *B. abortus* by itself is unable to traverse these cells.

Bacteria, such as *B. abortus* that are skillfully adapted to the intracellular milieu of a peripheral immune cell would have the ability to traverse the BBB by the “Trojan horse” mechanism (Dando et al., 2014). Many intracellular bacteria are able to infect the CNS by taking advantage of the physiological leukocyte traffic that traverses the BBB (Carson et al., 2006). Even at steady-state conditions, there are a low but significant number of monocytes and lymphocytes that incessantly patrol the CNS and that are able to cross brain capillaries with an intact BBB. This has created the analogy of the Trojan horse: in the same way that the wooden horse that carried hidden enemies into the walled city of Troy; phagocytes transport intracellular microbes through the BBB into the CNS. Likewise, although we have not observed any significant difference in the number of monocytes (infected or not) that migrate across the monolayer of HBMEC, *B. abortus* presence in the basolateral chambers of the transwell was scored as a consequence of migrating monocytes carrying viable bacteria. To our knowledge, this is the first experimental description of the mechanism whereby *B. abortus* is able to traverse the BBB. More importantly, our results indicate that these infected monocytes would be a source of infected bacteria for other cell types within the brain parenchyma such as microglia and astrocytes. It was recently described that *B. abortus* is capable of cell-to-cell spreading within a define cell type (either macrophages or Hela cells) by transforming the replicative *Brucella*-containing vacuole into a modified autophagic vacuole (Starr et al., 2012; Smith et al., 2016). Our experimental observations expand the possibilities of this bacterium to egress from one cell type and being able to infect another cell type thereby broadening its ability to reach different cell types in different organs.

In summary, results presented here describe the mechanism by which *B. abortus* can traverse the BBB and infect glial cells to generate an inflammatory response.

## Author contributions

MM, AR, and GG conceived and designed the experiments. MM, AR, MD, JR, and PB performed the experiments. MM analyzed the data and wrote sections of the manuscript. MD performed the infections with viable *B. abortus*. KK and VD supported the work with key suggestions and helped with data interpretation. GG supervised experiments, interpreted the data and wrote the manuscript. All authors reviewed the manuscript.

### Conflict of interest statement

The authors declare that the research was conducted in the absence of any commercial or financial relationships that could be construed as a potential conflict of interest.
